# Primary sclerosing epithelioid fibrosarcoma of kidney with variant histomorphologic features: report of 2 cases and review of the literature

**DOI:** 10.1186/s13000-015-0420-z

**Published:** 2015-10-09

**Authors:** Dilek Ertoy Baydar, Kemal Kosemehmetoglu, Oguz Aydin, Julia A. Bridge, Berrin Buyukeren, Fazil Tuncay Aki

**Affiliations:** Department of Pathology, Hacettepe University Hospital, Ankara, Turkey; Department of Pathology, Ondokuz Mayis University Hospital, Samsun, Turkey; Departments of Pathology/Microbiology, Pediatrics and Orthopaedic Surgery, Nebraska Medical Center, Omaha, NE USA; Department of Urology, Hacettepe University Hospital, Ankara, Turkey

**Keywords:** Sclerosing epithelioid fibrosarcoma, Kidney, EWSR1, CREB3L1, Translocation

## Abstract

The authors present two cases of primary sclerosing epithelioid fibrosarcoma (SEF) of the kidney. Both patients had a mass in the upper part of the left kidney without any primary extrarenal neoplastic lesions. Grossly, the tumors were solid masses both measuring 7.5 cm in the greatest diameter. Histologically, one of the lesions exhibited a predominantly lobular growth of round or oval small uniform epithelioid cells in variable cellularity. Circular zones of crowded tumor cells alternating with hypocellular collagenous tissue in a concentric fashion around entrapped native renal tubules were distinctive. The second case was distinctive with significant cytological atypia in the neoplastic cells and prominent reactive proliferations in the trapped renal tubules. Immunohistochemically, vimentin, bcl-2 and MUC4 were diffusely positive in both. They were negative for S-100 protein, CD34, and desmin, whereas CD99 were positive in one lesion. Fluorescence in situ hybridization assay using dual staining probes detected EWSR1-CREB3L1 fusion in each lesion, which is characteristic molecular findings of SEF. One patient presented widespread distant metastases at the time of diagnosis. In the other, no tumor deposits were detected other than primary. Both patients have been alive with 30 and 10 month follow-ups, respectively. These tumors are 6th and 7th cases of primary renal SEF in the literature confirmed by FISH study, which exhibit unique and remarkable histomorphologic features.

## Background

Sclerosing epithelioid fibrosarcoma (SEF) is a rare malignant mesenchymal tumor of soft tissues composed of cords, nests or sheets of relatively monotonous epithelioid cells within a collagenous background. It has recently been characterized by recurrent FUS-CREB3L1, FUS-CREB3L2 or EWS1-CREB3L1 translocations and immunohistochemical MUC4 expression [1–3]. The tumor occurs over a wide age spectrum at initial presentation without sex predilection. Most reported cases are in the soft tissue of extremities and limb girdles [4–7]. Primary SEF in visceral organs is exceedingly rare, with only a few cases reported.

Here, we present two cases of SEF with primary renal origin bearing a EWS1-CREB3L1 gene fusion, as supported by MUC4 immunostaining and fluorescence in situ hybridization (FISH). To our knowledge, these are the sixth and seventh genetically confirmed cases of SEF reported in the kidney following very recently described primary renal SEF(s) by Arbajian et al., Argani et al. and Ohlmann, et al. [1, 8, 9]. Some of their morphologic features are unique and deserve to be noted for full characterization of this entity in a new environment.

## Case presentation

Two cases of SEF that were found to harbor EWSR1-CREB3L1 fusion were encountered in the diagnostic practice of the authors. The clinical records were retrieved for analysis, and all available pathologic materials were reviewed.

### Immunohistochemistry

For immunohistochemical labeling, a polymer detection system (Leica, DS9800) and the BOND-MAX automated immunostainer was used. The standard antibodies consumed, vendors, and dilutions were summarized in Table 1.

**Table 1:** Features of the antibodies used for the immunohistochemical stains

Antigen	Clone, dilution, source
Pan-Cytokeratin	AE1/AE3, 1:200, Leica, Newcastle/UK
Epithelial Membrane Antigen	E29, 1:300, Biocare, Concord/CA
PAX8	PAX8, 1:100, Biocare, Concord/CA
WT1	6 F-H2, 1:40, DBS, Pleasenton/CA
ER	EP1, 1:100, Genemed, San Francisco/CA
PR	SP2, 1:1000, Thermo, Fremont/CA
CD34	QBEND/10, 1:100, DBS, Pleasenton/CA
SMA	1A4, 1:1000, NeoMarkers, Fremont/CA
Desmin	D33, 1:50, Biocare, Concord/CA
S100 protein	Z0311, 1:6000, DAKO, Glostrup/Denmark
GFAP	GA-5, 1:50, Thermo, Fremont/CA
Bcl2	Bcl-2-100, 1:80, Invitrogen, Paisley/UK
CD99	HO36-1.1, 1:100, Thermo, Fremont/CA
MUC4	1G8, 1:50, Invitrogen, Paisley/UK
HMB45	HMB45, 1:25, DBS, Pleasenton/CA
Melan-A	A103, 1:100, Thermo, Fremont/CA
INI1	25, 1:50, Zeta, Arcadia/CA

### Fluorescence In Situ Hybridization (FISH)

FISH analysis was performed on representative 4–5 μm thick unstained formalin-fixed, paraffin-embedded tissue sections of the tumor samples of each case. For characterization of the possible underlying gene rearrangement or fusion gene events, the following FISH probe sets were utilized on both cases: Vysis LSI EWSR1 (22q12) and Vysis LSI FUS (16p11) Dual Color Break Apart Probes (Abbott Molecular, Inc., Des Plaines, IL) and *EWSR1* and *CREB3L1* spanning probe sets using cocktails of BAC clones (RP11-945 M21 and RP11-1126O13, and RP11-1014A16, RP11-1106 J11 and RP11-481I24 respectively) selected on the basis of their location per the UCSC Human Genome Browser [http://genome.ucsc.edu/cgi-bin/hgGateway] and obtained from BAC/PAC Resources Center (Children’s Hospital Oakland Research Institute, Oakland, CA, USA).

Hybridization studies using the Vysis LSI Dual Color Break Apart Probes for the assessment of rearrangement of the *FUS* and *EWSR1* loci were performed following the manufacturer’s instructions (Abbott Molecular, Inc., Des Plaines, IL). With respect to the custom spanning probes, each BAC clone was directly labeled by nick translation with either Spectrum Green- or Spectrum Orange-dUTP per the manufacturer’s protocol (Abbott Molecular, Inc., Des Plaines, IL). An amount of 3 ug of DNA for each probe or 1.5 ug for each of two probes were combined. All nick translation reagents were then multiplied by the total ug of DNA used in the cocktail. Amounts of 200 ng of each probe were hybridized to the target DNA and blocked with approximately 15 fold excess of a combination of Human Cot-I DNA (Invitrogen, Carlsbad, CA) and human placental DNA.

Prior to hybridization, the slides were pretreated at room temperature in 0.2 N HCl for 20 min, washed in water for 3 min, incubated at 80 °C for 25 min in VP 2000 Pretreatment Reagent (Abbott Molecular, Inc., Des Plaines, IL) and then washed again in water for 3 min. Subsequently, the slides were incubated for 15 min at 37 °C in protease solution [25 mg of protease in 50 ml of protease solution (Abbott Molecular, Inc., Des Plaines, IL), washed in 1 × PBS at room temperature for 5 min and then dehydrated in gradient ethanol (75, 85, and 100 %) at room temperature for 1 min each and air-dried. After the cells and probes were co-denatured at 80 °C for 10 min and incubated overnight at 37 °C using the HYBrite™ system (Abbott Molecular, Inc., Des Plaines, IL), post-hybridization washing was performed in 2 × SSC/0.1 % NP-40 at 72 °C for 2 min, followed by 2 × SSC/0.1 % NP-40 at room temperature for 1 min. The slides were then counterstained with DAPI II (Abbott Molecular, Inc., Des Plaines, IL). To confirm correct mapping, optimal signal strength, and lack of cross-hybridization, each probe set was also hybridized to metaphase cell preparations of karyotypically normal peripheral blood lymphocytes before proceeding with analysis of the patient samples.

The cutoff level for scoring a specimen as positive for a rearrangement of the *FUS* or *EWSR1* locus or as positive for an *EWSR1/CREB3L1* fusion was >15 % of the cells evaluated. Images were prepared using the Cytovision Image Analysis System (Applied Imaging, Santa Clara, CA). For each probe set, 100–200 interphase nuclei with strong and well-delineated signals were examined.

### Case 1

**Fig. 1:**
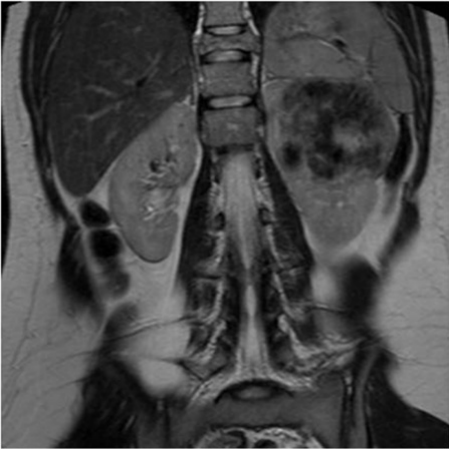
Case #1. Computerized tomography scan showing a large tumor in the left kidney

The patient was a 16-year-old girl who presented at the urology clinics with pain on the left side of the abdomen radiating to the back. Her past medical history was insignificant. Computerized axial tomography scans of the chest, abdomen, and pelvis revealed a 70 × 70 × 60 mm left renal mass (Fig. 1), bilateral pulmonary nodules (the largest being 16 mm in diameter), and widespread bone metastases in vertebrae, sacrum and left femoral head. A biopsy from the tumor in the kidney through laparotomy was performed followed by left radical nephrectomy after the diagnosis of malignancy.

**Fig. 2:**
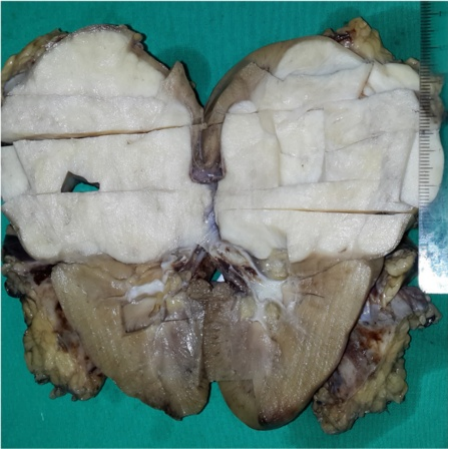
Case #1. Solid white tumor filling out the upper half of the kidney

**Fig. 3:**
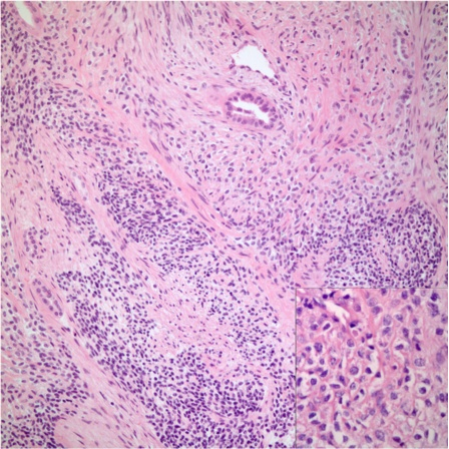
Case #1. Alternating hypercellular and a hypocellular areas of neoplastic cells with monomorphic ovoid nuclei and indistinct pale to clear cytoplasm (H&E x 200; inset: H&E x 400)

**Fig. 4:**
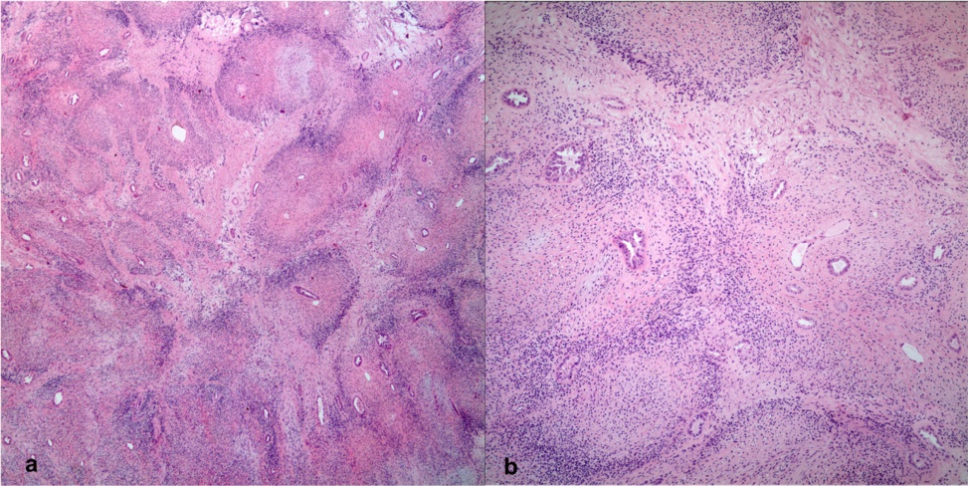
Case #1. **a** Lobular/micronodular pattern (H&E x 40). **b** Concentric hypocellular inner, hypercellular outer zone around entrapped renal tubules (H&E x 100)

**Fig. 5:**
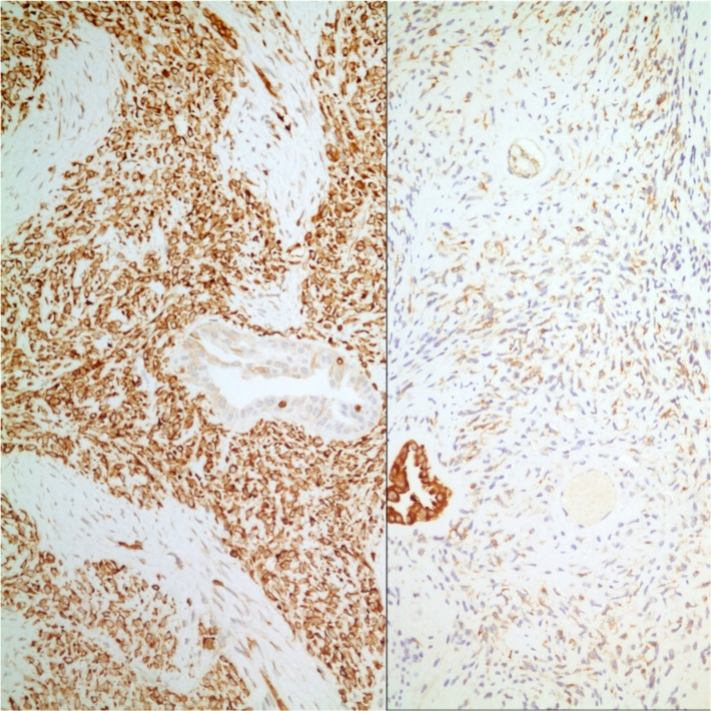
Case #1. Strong bcl-2 and weak EMA expression by neoplastic cells (*Left*: Immunohistochemistry, anti-bcl-2 Ab x 200; *Right*: Immunohistochemistry, anti-EMA Ab x 200)

**Fig. 6:**
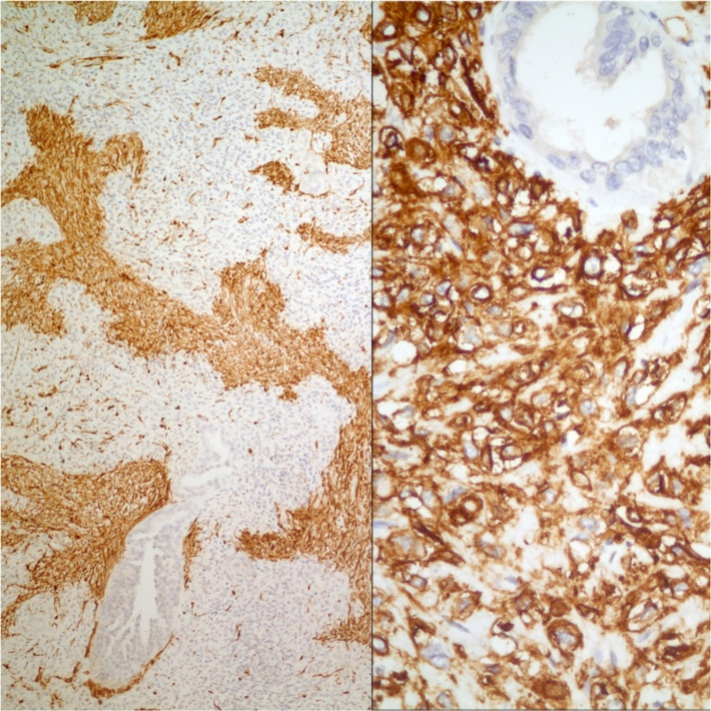
Case #1. SMA stains myofibroblasts between neoplastic lobules and MUC4 stains neoplastic cells (*Left*: Immunohistochemistry, anti-SMA Ab x 100; *Right*: Immunohistochemistry, anti-MUC4 Ab x 400)

**Fig. 7:**
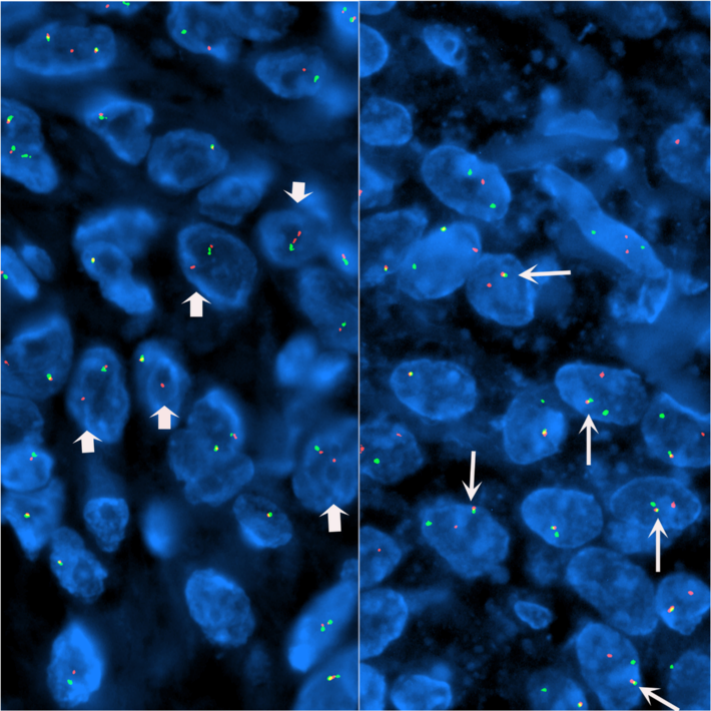
Case #1. An *EWSR1* Break Apart probe set and a custom probe set spanning the *EWSR1* (Spectrum Orange) and the *CREB3L1* (Spectrum Green) loci demonstrating loss of the Spectrum Green labeled probe signal that flanks the 3’ (telomeric) side of the *EWSR1* locus (short arrows, left panel) and a single fusion signal (long arrows, right panel)

Tumor occupied the entire upper half of the kidney, extended into the renal sinus, was a solid white unencapsulated lesion with sharp borders, measuring 7.5 × 7 × 7 cm in size (Fig. 2). Microscopical sections showed diffuse infiltration of the neoplastic tissue in kidney parenchyma separating normal renal elements from each other. The neoplastic cells were small, monotonous and epithelioid with clear to pale eosinophilic cytoplasm, and were arranged in single files, cords, nests or irregular aggregates in collagenous matrix (Fig. 3). Nuclei were generally round to oval, with indistinct nucleoli. Hypercellular areas alternated randomly with hypocellular densely hyalinized or at times myxoid stroma. Additionally, a peculiar lobular organization was noted in many regions where a renal tubule in the center was surrounded by concentric inner hypo and outer hypercellular zones of neoplastic cells (Fig. 4a and b). These lobules were separated from each other by myofibroblasts. Tubules entrapped in the tumor were lined by single layered Pax-8 positive cuboidal cells without atypia, some showed shallow papillary hyperplasia and rare mitosis. Mitotic rate was 1/10 hpf in the neoplasm and there were occasional areas of necrosis in the tumor. Hypercellular areas occasionally contained vague nodules of collagen mimicking those seen in hyalinizing spindle cell tumor with giant rosettes (HSCTGR). The surrounding kidney showed no specific pathologic changes. By immunohistochemistry, neoplastic cells were immunoreactive diffusely and strongly for vimentin, bcl-2 and CD99; EMA labelled them in a weak and patchy fashion (Fig. 5). They were negative for pan-cytokeratin, Pax-8, WT-1, CD34, S-100, GFAP, Melan-A, HMB-45, desmin, and estrogen and progesterone receptors. Smooth muscle actin (SMA) stained myofibroblastic cells in-between the neoplastic lobules (Fig. 6). INI-1 was preserved. Then, an immunohistochemical stain for MUC4 was performed which showed strong positivity throughout the tumor (Fig. 6). Finally, FISH analysis with the EWSR1 Break Apart probe revealed loss of one copy of the Spectrum Green labeled probe flanking the 3’ (telomeric) side of the *EWSR1* gene as well as the presence of a single fused *EWSR1/CREB3L1* signal (represented by a juxtaposed orange signal and green signal) consistent with the presence of an unbalanced der(22)t(11;22)(p11;q12) (Fig. 7), confirming the diagnosis of sclerosing epithelioid fibrosarcoma. FISH study was negative for a rearrangement of the *FUS* gene locus.

Patient was given chemotherapy with multiple agents. She is alive with disease after 30 month follow-up.

### Case 2

The patient was a 57-year-old woman who was investigated for cholelithiasis due to dyspeptic complaints. During abdominal ultrasonography, she was found to have a left renal mass incidentally. The CT scan showed that the tumor was located at the upper pole of the kidney and was measured 6 cm in the largest diameter. Her past medical history was unremarkable except for hypertension, congestive heart failure and arrhythmia. Serum and urine tests were within the normal limits. The patient subsequently underwent open left partial nephrectomy.

**Fig. 8:**
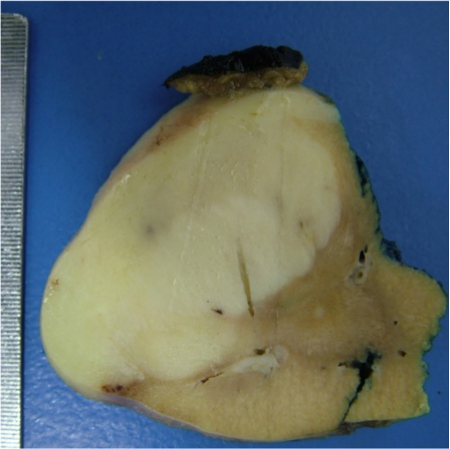
Case #2. Gross photograph of the kidney in patient no. 2 shows a well-defined solid mass that extends to surgical margin of the partial nephrectomy specimen

**Fig. 9:**
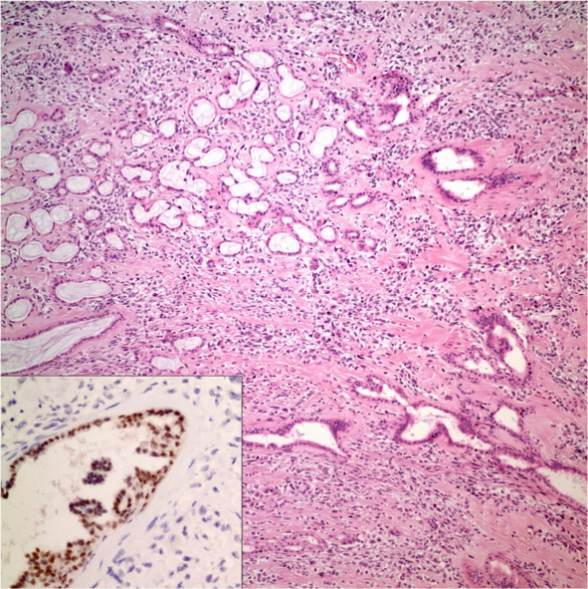
Case #2. Proliferating native renal tubules inside the tumor giving an appearance of a biphasic lesion (H&E x 100). Inset shows that hyperplastic tubules maintain nuclear pax-8 (H&E x 40)

**Fig. 10:**
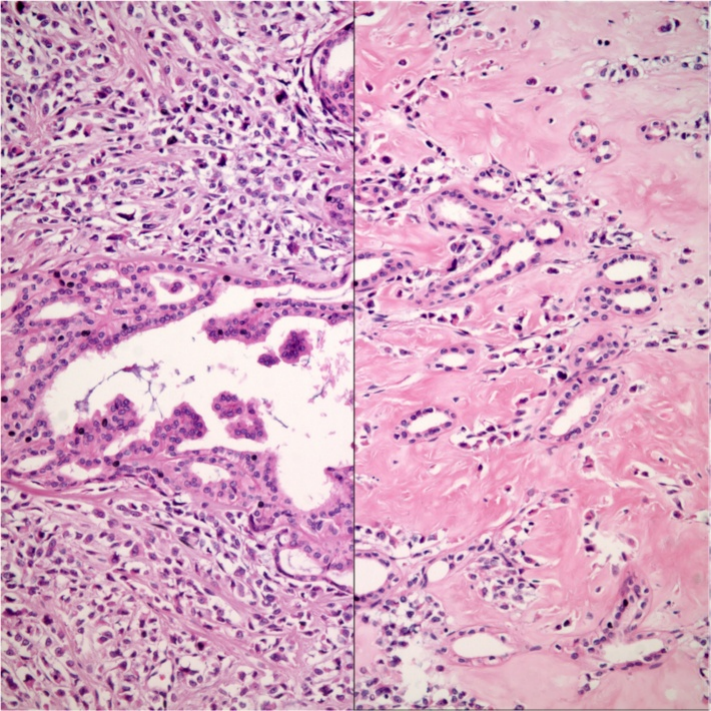
Case #2. Hyper and hypocellular regions (*Left*: H&E x 200; *Right*: H&E x 200)

**Fig. 11:**
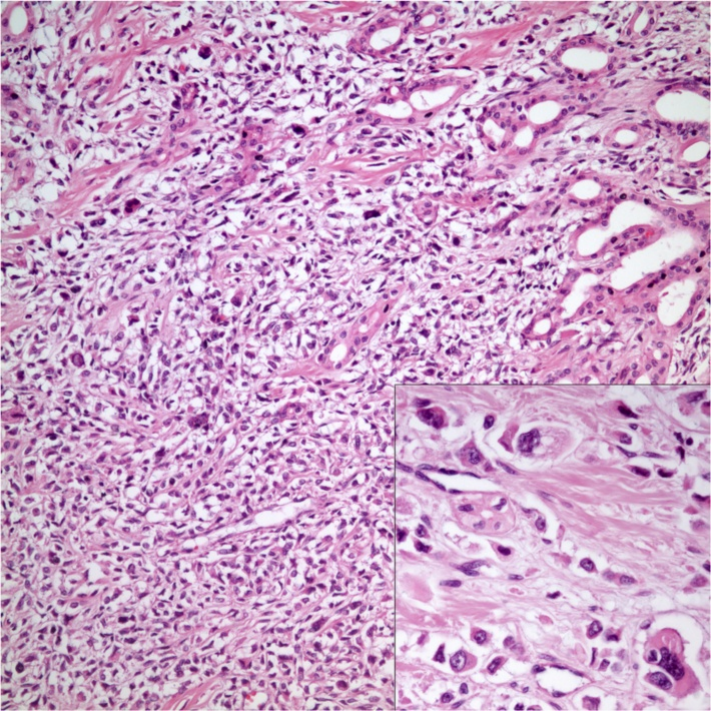
Case #2. Spindle cells forming short fascicles vaguely (H-E x 200). Inset highlights marked pleomorphism among the neoplastic cells (H-E x 400)

**Fig. 12:**
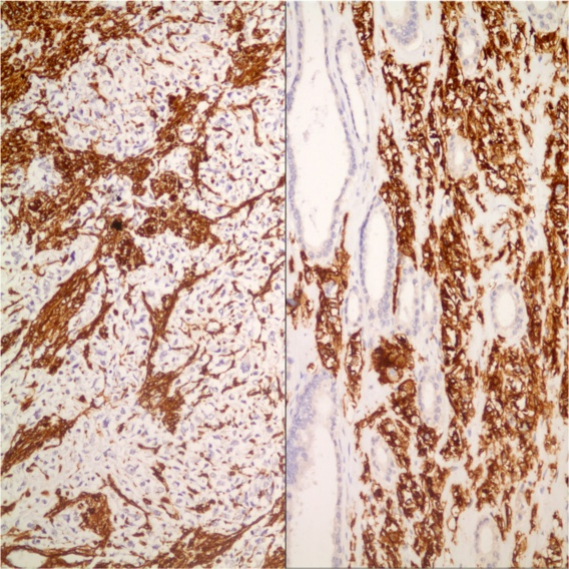
Case #2. SMA stains interspersed myofibroblasts and MUC4 labels neoplastic cells (*Left*: Immunohistochemistry, anti-SMA Ab x 200; *Right*: Immunohistochemistry, anti-MUC4 Ab x 200)

**Fig. 13:**
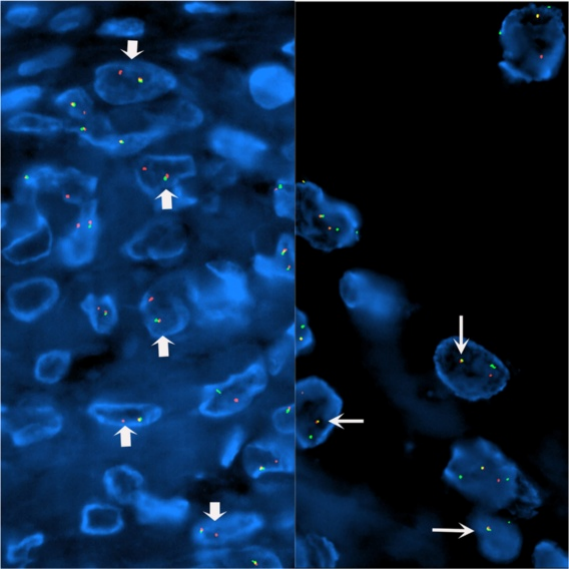
Case #2. FISH patterns similar to Case 1. *EWSR1* break apart with loss of one copy of the Spectrum Green labeled probe flanking the 3’ (telomeric) side (left panel) and *EWSR1/CREB3L1* fusion seen by a juxtaposed orange signal and green signal (right panel)

On gross examination, the specimen had an unencapsulated, solid - white firm tumor with irregular borders, measuring 7.5 × 5.5 × 4 cm (Fig. 8). Histologic examination revealed that tumor contained abundant entrapped native renal tubules throughout as in the first case, mimicking a biphasic neoplasm (Fig. 9). These tubules were hyperplastic and proliferating, were of various size and shapes, some being cystic or leaf-like, and lined by single layered cuboidal or occasionally flattened cells, all expressing nuclear Pax-8. Intraluminal papillary projections were common, but without cytologic atypia. Neoplastic component had a variable cellularity (Fig. 10). Hypocellular regions contained abundant hyalinized collagen and some showed myxoid change. Hypercellular areas were divided into anastomosing compartments by sclerotic collagen bands. Neoplastic cells were polygonal epithelioid or plump spindle type forming short fascicles (Fig. 11). Cytoplasm was clear or eosinophilic. Cellular pleomorphism was prominent with scattered bizarre and hyperchromatic nuclei, intranuclear inclusions were evident in some. Tumor abutted lining urothelium of renal pelvis without ulceration and formed large hypocellular sheets of short spindle cells in myxoid matrix encircling pelvic wall. There were rare foci of necrosis. Mitotic rate ranged 5–10/10 hpf. Immunohistochemical profile of tumor was similar to the first case including strong and diffuse MUC4, vimentin and bcl-2, weak EMA expression (Fig. 12) except negative immunoreactivity for CD99. SMA showed frequently scattered myofibroblasts between the tumor cells. FISH demonstrated an unbalanced der(22)t(11;22)(p11;q12), as in the case #1 (Fig. 13). A *FUS* gene rearrangement was not identified.

The lesion occupied both renal cortex and medulla, and invaded into peripelvic and perirenal fat tissue. As one of the surgical margins was in continuation with the tumor, the patient underwent left radical nephrectomy which showed 1 cm residual mass. No adjuvant treatment was given. Patient is alive without local or distant recurrence 10 months after surgery.

## Discussion

Sclerosing epithelioid fibrosarcoma (SEF) was first described by Meis-Kindblom et al. in 1995 [6]. The most SEFs are deep seated lesions with a wide age range, but typically occurring in middle aged adults [4, 6]. The most frequent sites of involvement are deep soft tissues of lower extremities or limb girdle followed by trunk, upper extremities, and head and neck region, although primary SEF of unexpected locations such as bone, retroperitoneum, and pelvis were also described as case reports or small series [10–13]. Primary SEF in visceral organs is exceedingly rare, with only a single case reports in the liver [12], the lower gastrointestinal tract [11], the ovary [14], and the pancreas [10]. Occurrence of primary renal SEF was proven by Argani et al. who have recently reported 2 well-documented cases with rearrangement involving EWSR1 and CREB3L1 genes [8] (Table 2). There are also 3 additional cases in the literature described as primary renal SEFs which were shown to express MUC4 [1, 9]. Two of these had rearranged EWSR1 with undocumented fusion gene partner.

**Table 2:** Summary of reported cases of primary renal SEF

Authors	Age (years)	Gender	Largest Diameter /Laterality	Clinical presentation	FISH Findings	Therapy	Metastases	Outcome (mo)
Arbajian, et al. [1]	41	Female	9 cm/NS	NS	EWSR1 Del 3′	NS	Bone and lung	DOD (22)
Argani, et al. [8]	17	Male	25 cm/L	Left flank, back and abdominal pain, weight loss, dysuria, and decreased appetite	EWSR1-CREB3L1 fusion	Surgery + RT	Rib, vertebrae, epidural spinal cord and liver	DOC (1)
Argani, et al. [8]	61	Female	5 cm/L	Rib pain	EWSR1-CERB3L1 fusion	Surgery	Ribs, bone, lung and lymph nodes	AWD (6)
Ohlmann, et al. [9]	24	Female	22 cm/R	NS	No results	Surgery + RCT	Lungs and vertebrae	DOD (82)
Ohlmann, et al. [9]	43	Male	4.2 cm/R	Incidental	EWSR1 split	Surgery	None	ANED (8)
Ertoy Baydar, et al. (present case)	16	Female	7.5 cm/L	Abdominal pain radiating to back on the left	EWSR1-CREB3L1 fusion	Surgery + CT	Lungs, vertebrae, sacrum and left femoral head	AWD (30)
Ertoy Baydar, et al. (present case)	57	Female	7.5 cm/L	Incidental	EWSR1-CREB3L1 fusion	Surgery	None	ANED (10)

*NS* Not specified, *L* Left kidney, *R* Right kidney, *RT* Radiotherapy, *CT* chemotherapy, *RCT* Radiochemotherapy, *DOD* Died of disease, *DOC* Died of complications related to disease treatment, *AWD* Alive with disease, *ANED* Alive with no evidence of disease

SEF is a difficult diagnosis in visceral organs because of its rarity and its epithelioid appearance, closely mimicking carcinomas. It is characterized by a proliferation of epithelioid cells arranged in nests and cords in a densely hyalinized stroma (Table 3). Neoplastic cells are positive for vimentin, bcl-2, MUC4, weakly and focally for epithelial membrane antigen (EMA), and are negative for broad spectrum cytokeratins, smooth muscle actin, desmin, CD34, S-100 protein, HMB45 and melan-A [2, 4, 15]. MUC4 itself has been recently reported as highly specific for the diagnosis of SEF [2]. Strong CD99 expression is seen in some cases. Most common genetic alteration described in pure SEFs is EWSR1-CREB3L1 fusion [1]. Basic histomorphological differentials of SEF will be a variety of tumors with epithelioid and sclerotic features, mainly primary or metastatic carcinoma. Epithelioid angiomyolipoma, metanephric stromal tumor and synovial sarcoma are the other considerations in the differential diagnosis, as well as sclerosing clear cell sarcoma of the kidney (CCSK) being the most challenging. A battery of immunohistochemical stains will help for further characterization in most circumstances, however SEF and sclerosing type CCSK reveal both similar morphology and immunohistochemical findings Argani et al. suggest that some cases reported as sclerosing clear cell sarcoma of the kidney (CCSK) in the literature might in fact represent SEF [8].

**Table 3:** Morphology of SEF

Typical features	
Macroscopy	Large, homogeneously white or white–tan, lobulated, and hard tumors
Cell size and shape	Small to medium-sized plump to epithelioid cells
Cytoplasm	Scant clear or eosinophilic cytoplasm
Nuclei	Oval to slightly elongate angulated nuclei with finely speckled chromatin
Cellularity	Variable within the neoplasm
Atypia	Mild
Matrix	Densely sclerotic, areas of metaplastic bone in some
Pattern of cellular arrangement	Small clusters, nests and anastomosing cords
Hybrid morphology	Areas of LGFMS or nodules of collagen reminiscent of HSCTGR in some
Immunohistochemistry	MUC4 ++, EMA +/−, CD99 +/−, bcl2 +/−, pan-keratin -, S100/HMB45/MelanA -
FISH	EWSR1 (or rarely FUS) rearrangements
Previously unrecognized features in renal SEF	
Current case 1	A lobular or micronodular architecture due to neoplastic cells surrounding entrapped renal tubules in a concentric fashion
	Zonation in the neoplastic lobules around tubules with inner hypocellular and outer hypercellular appearance
Current case 2	Exuberant epithelial hyperplasia and small gland budding in the entrapped native renal tubules, mimicking MEST
	High grade cytologic atypia

*LGFMS* Low grade of fibromyxoid sarcoma, *HSCTGR* Hyalinizing spindle cell tumor with giant rosettes, *MEST* Mixed epithelial and stromal tumor of kidney

SEF and low-grade fibromyxoid sarcoma (LGFMS) are thought to be related members in the *fibrosing fibrosarcoma family* [4]. Classic variants of LGFMS with or without giant rosettes constitute one end of the spectrum, characterized by a protracted clinical course and a low metastatic rate, whereas SEF or cellular variants of LGFMS constitute the other end which appears to be more aggressive. LGFMS may have SEF-like areas, and vice versa occurs in SEF lesions. Both tumors are labelled by immunohistochemical MUC4 staining, 99–100 % in LGFMS and 78 % in SEF, respectively [2, 16]. Furthermore, a genetic link between sclerosing epithelioid fibrosarcoma and low-grade fibromyxoid sarcoma has been suggested [17]. Although majority (>90 %) of LGFMS and hybrid LGFMS/SEF harbor t(7;16)(q33;p11) chromosome translocation resulting in FUS-CREB3L2 gene fusion, rare LGFMS carrying EWSR1-CREB3L1 gene fusion with t(11;22)(p11;q12) chromosome translocation was also found [18]. There have been 5 cases of primary LGFMS described in kidney or renal pelvis so far, one in a 6 year old child, others in adults [19–23].

We report 2 distinctive, clinically malignant, renal SEF bearing EWSR1-CREB3L1 fusions through unbalanced translocation with unique histomorphologic features. The common finding in both was the diffuse infiltration of the tumor among renal tubules and glomeruli filling out the space in-between them. Numerous tubules and also glomeruli were seen entrapped throughout the tumors. In case #1, neoplastic cells surrounded these tubules in a concentric onion-skin like pattern giving a lobular or micronodular architecture to the lesion in many areas. Additionally, the neoplastic sleeves around tubules revealed a zonation pattern with inner hypocellular and outer hypercellular appearance. This was a wide-spread occurrence involving also the deeper parts of the tumor, so differed from what is seen in CCSK or metanephric stromal tumor where neoplasm encircles native tubules at the tumor infiltration borders in a limited extent. No angiodysplasia or juxtaglomerular hyperplasia was found in our case.

The second case presented in this study varied from the first one in that the entrapped renal tubules exhibited prominent papillary hyperplasia and small gland budding which gave a biphasic appearance to the lesion. They were lined by cuboidal or flattened single layered epithelium devoid of atypia, some were cystic, and some looked fibroadenomatous and leaf-like due to compression by the interstitial neoplastic cells. It is quite likely that we would have diagnosed this lesion as malignant mixed epithelial and stromal tumor of kidney (MEST) only a few years ago when typical genetic alterations or MUC4 staining characteristic of this tumor type were unknown yet. In fact, we suspect that other examples of renal SEF may have been reported in the literature with an assignment as malignant MEST. Suzuki et al. [24] reported a malignant MEST in a male with a prostatic adenocarcinoma under anti-androgen treatment, that was composed of atypical small round cells with a high nuclear cytoplasmic ratio without necrosis and pleomorphism, and also focal spindle cells without cytologic atypia in a gradual transition to small round cells. Immunohistochemically, tumor was bcl-2 and CD99+, SMA and S-100 -. *SYT-SSX1* and *SYT-SSX2* chimeric transcript were not identified. We suspect that at least this published case may represent the entity reported herein.

Another feature of the second case of this study that varied from the first one and from 5 cases reported previously was the presence of high grade cytologic atypia with noteworthy pleomorphism and scattered bizarre tumor cells. Most SEFs are cytologically uniform tumors with scanty mitoses; however, it is stated that foci of pleomorphism -particularly in hybrid SEF and LGFMS cases- and high mitotic activity can rarely be encountered [4, 7, 17].

SEF is frequently characterized by aggressive clinical behavior [4, 6]. One or more local recurrences occur in approximately 50 % of cases, with metastatic spread being reported in more than 40 % of cases, most often affecting the pleura, lungs, bone and central nervous system. All of the cases reported previously except one and our first case were already metastatic at the time of presentation. As accurate recognition of SEF is important for appropriate patient management, we believe more cases need to be documented so that its full histomorphologic spectrum is uncovered.

## Conclusions

Sclerosing epithelioid fibrosarcoma (SEF) is a rare soft tissue tumor that can occur in the kidney as a primary malignancy. Its misdiagnosis with other entities which are better known to develop in the kidney is a strong possibility as it has been only recently described and is unfamiliar to the pathologists. SEF is a neoplasm with variant morphological features that may overlap with many lesions confusingly. Immunohistochemistry and molecular studies that disclose the characteristic genetic alterations are crucial for accurate recognition.

## Consent

Written informed consent was obtained from the parent (case #1) and the patient (case #2) for publication of this Case Report and any accompanying images. A copy of the written consent is available for review by the Editor-in-Chief of this journal.

## Abbreviations

SEF: Sclerosing epithelioid fibrosarcoma
FISH: Fluorescence in situ hybridization
HSCTGR: Hyalinizing spindle cell tumor with giant rosettes
CT: Computerized tomography
CCSK: Clear cell sarcoma of the kidney
LGFMS: Low-grade fibromyxoid sarcoma
MEST: Mixed epithelial and stromal tumor of kidney
